# Prediction of Anti-rheumatoid Arthritis Natural Products of Xanthocerais Lignum Based on LC-MS and Artificial Intelligence

**DOI:** 10.2174/0113862073282138240116112348

**Published:** 2024-01-30

**Authors:** Hao Qian, Zhibin Xiao, Lei Su, Yaqiong Yang, XiangYang Tian, Xiaoqin Wang

**Affiliations:** 1 College of Pharmacy, Inner Mongolia Medical University, Hohhat, China

**Keywords:** Xanthocerais lignum, rheumatoid arthritis, artificial intelligence, machine learning, LC-MS, active natural products

## Abstract

**Aims:**

Employing the technique of liquid chromatography-mass spectrometry (LC-MS) in conjunction with artificial intelligence (AI) technology to predict and screen for anti-rheumatoid arthritis (RA) active compounds in Xanthocerais lignum.

**Background:**

Natural products have become an important source of new drug discovery. RA is a chronic autoimmune disease characterized by joint inflammation and systemic inflammation. Although there are many drugs available for the treatment of RA, they still have many side effects and limitations. Therefore, finding more effective and safer natural products for the treatment of RA has become an important issue.

**Methods:**

In this study, a collection of inhibitors targeting RA-related specific targets was gathered. Machine learning models and deep learning models were constructed using these inhibitors. The performance of the models was evaluated using a test set and ten-fold cross-validation, and the most optimal model was selected for integration. A total of five commonly used machine learning algorithms (logistic regression, k-nearest neighbors, support vector machines, random forest, XGBoost) and one deep learning algorithm (GCN) were employed in this research. Subsequently, a Xanthocerais lignum compound library was established through HPLC-Q-Exactive-MS analysis and relevant literature. The integrated model was utilized to predict and screen for anti-RA active compounds in Xanthocerais lignum.

**Results:**

The integrated model exhibited an AUC greater than 0.94 for all target datasets, demonstrating improved stability and accuracy compared to individual models. This enhancement enables better activity prediction for unknown compounds. By employing the integrated model, the activity of 69 identified compounds in Xanthocerais lignum was predicted. The results indicated that isorhamnetin-3-*O*-glucoside, myricetin, rutinum, cinnamtannin B1, and dihydromyricetin exhibited inhibitory effects on multiple targets. Furthermore, myricetin and dihydromyricetin were found to have relatively higher relative abundances in Xanthocerais lignum, suggesting that they may serve as the primary active components contributing to its anti-RA effects.

**Conclusion:**

In this study, we utilized AI technology to learn from a large number of compounds and predict the activity of natural products from Xanthocerais lignum on specific targets. By combining AI technology and the LC-MS approach, rapid screening and prediction of the activity of natural products based on specific targets can be achieved, significantly enhancing the efficiency of discovering new bioactive molecules from medicinal plants.

## 1 INTRODUCTION

RA is a chronic systemic autoimmune disease characterized by erosive, symmetrical polyarthritis. Its typical features include synovial cell proliferation, synovitis, cartilage damage, and periarticular bone destruction [1]. Currently, the drugs commonly used in clinical practice for RA treatment mainly include nonsteroidal anti-inflammatory drugs, disease-modifying antirheumatic drugs, glucocorticoids, and biologics [2]. However, these drugs have limitations such as serious adverse effects, slow onset of action, and high cost. Traditional medicines, such as Chinese and Mongolian medicines, have a wide range of sources, diverse categories, and minimal adverse effects. Many of their components have biological activities such as anti-inflammatory and immune modulation. Therefore, exploring more effective and safer natural products from traditional medicines has become a research hotspot for RA treatment.

Xanthocerais lignum is the dried stem or branch of the *Xanthoceras sorbifolia* Bunge. tree in the Sapindaceae family. It is recorded in classical Mongolian and Tibetan pharmacopoeias such as “*Ren Yao Bai Jing Jian*,” “*Wu Wu Meng Yao Jian*,” “*Jing Zhu Ben Cao,*” and “*Meng Yao Zhi*.” It has the effects of clearing heat, reducing swelling, and relieving pain. Clinically, it is mainly used to treat RA [3-5]. Traditional efficacy and modern pharmacological research have both shown that Xanthocerais lignum has great potential as an anti-RA agent, but its active natural products that exert anti-RA activity have yet to be elucidated, which limits its further development and utilization. By gaining a comprehensive understanding of the active constituents of Xanthocerais lignum, it is possible to more effectively utilize these natural products for the treatment of diseases such as RA. In comparison to conventional chemically synthesized drugs, natural products exhibit a wider range of biological activities and fewer adverse effects, thus rendering them a safer and more efficacious treatment option. These natural products hold the potential to serve as novel drug targets, offering new insights and directions for the development of safer and more effective therapies for RA and other diseases.

Fibroblast-like synoviocytes (FLS) are a key component of proliferating synovium, and their excessive proliferation, impaired apoptosis, enhanced invasiveness and migration abilities can promote FLS accumulation in joints, leading to angiogenesis, inflammatory cytokine secretion, neovascularization and cartilage degeneration, ultimately exacerbating the progression of RA [6, 7]. Therefore, regulating FLS proliferation, apoptosis, invasiveness, and migration processes may be a promising RA treatment strategy. In our previous study, we used network pharmacology to predict that Xanthocerais lignum may exert anti-RA effects by mediating the PI3K/AKT signaling pathway. Therefore, in this study, we opted to screen the active ingredients of targets related to processes such as proliferation, apoptosis, invasion, and migration in the PI3K/AKT pathway.

The field of natural product pharmacology and active ingredient research has seen an increasingly widespread application of artificial intelligence (AI) technology. AI is fundamentally a data-driven approach that can automatically learn from large datasets and be used for predicting new data. Liquid chromatography-mass spectrometry (LC-MS) is an efficient analytical method that can be employed to rapidly identify the natural products in plants. In this study, we utilized AI to learn from a vast number of compounds and predict the activity of natural products from Xanthocerais lignum against specific targets. By combining AI with LC-MS, it is possible to rapidly screen and predict the activity of natural products against specific targets, greatly enhancing the efficiency of discovering novel bioactive natural products from medicinal plants.

## 2 MATERIALS AND METHODS

### 2.1 Data Preparation

After standardizing the names of each target using Uniprot (www.uniprot.org), relevant active molecules were retrieved from ChEMBL (www.ebi.ac.uk/chembl/) using the search terms “PI3K”, “AKT,” “Bcl2”, “EGFR,” “IGF1R”, “FAK” and “IRAK4”. The data was then cleansed using the following criteria: (1) selection of compounds with experimental activity types Kd, Ki, IC_50_, EC_50_; (2) removal of duplicate and compounds without experimental activity values or SMILES; (3) conversion of all compound activity values to nM units. After data cleansing, labels were added to the data where experimental activity values ≤ 1000 nM were defined as active molecules (label: 1), while experimental activity values > 1000 nM were labeled as non-active molecules (label: 0). Random stratified sampling was used to divide each target's dataset into training and testing sets in an 8:2 ratio. The training set is a group of data used to train the model. By learning from the data in the training set, the machine learning algorithm can generate a model that can be used to predict unknown data. The testing set is a group of data used to evaluate the model. By applying the model to the testing set data, the accuracy and effectiveness of the model can be evaluated [8]. The details of the compounds in each target data set are shown in Table **S1-S7**.

### 2.2 Machine Learning

#### 2.2.1 Molecular Characterization

Using Rdkit [9], molecular structures were generated based on the SMILES for each target's dataset. On this basis, 208 molecular descriptors [10], 1024 Morgan fingerprints [11], and 167 MACCS keys [12] were generated, totaling 1399 molecular features, to characterize the molecules. Molecular descriptors are a method of representing molecules as numerical features, typically using chemical structures and related features such as hydrogen bond acceptor/donor numbers, ring types, molecular weight, *etc*. Morgan fingerprints are a method of representing the position of molecules by hashing the molecule graph and generating a fixed-length numeric vector, where each number represents the presence of a particular chemical environment in the molecule. MACCS keys (Molecular ACCess System keys) are a method of representing key features of molecules, generating a fixed-length binary fingerprint using a set of pre-defined 166 key features, such as coordination number, ring type, *etc*. These molecular features can be used as input for machine learning algorithms to predict the activity and efficacy of molecules.

#### 2.2.2 Machine Learning Model Construction

Before constructing the model, data preprocessing was applied to improve the accuracy and stability of the model: (1) removal of molecules with a molecular weight greater than 1000; (2) removal of molecules containing missing values; (3) normalization of molecular descriptors. After data preprocessing, a machine learning model was constructed using 1399 molecular features as independent variables (X) and molecular activity labels as dependent variables (Y). Five common machine learning algorithms were used for each target dataset: logistic regression (LR), k-nearest neighbor (KNN), support vector machine (SVM), random forest (RF), and XGBoost. LR is a classification technique that uses linear regression and the Sigmoid function. It is mainly used for binary classification problems. The basic idea is to predict the result of a linear function through linear regression, then map it to the Sigmoid function to obtain a probability value and determine the category of the sample based on the probability value [13]. KNN is an instance-based learning method commonly used for classification and regression problems. The basic idea is that if most of the k most similar samples (i*.e*., the closest samples in feature space) belong to a certain category, then this sample also belongs to that category [13]. SVM is a binary classification model. The basic idea is to solve an optimization problem to determine the decision boundary (also known as the support vector) and divide the training samples into two categories. This decision boundary is a straight line or hyperplane that maximizes the distance (also known as margin) between the two classes of samples. The SVM model can find the decision boundary in a high-dimensional space by using a kernel function, making it very suitable for handling nonlinear problems [13]. RF is an ensemble learning model based on decision trees. The basic idea is to predict the target variable by randomly selecting training samples and using decision trees and then combining the results statistically to construct the final prediction. When building an RF model, multiple decision trees are constructed simultaneously and independently, and data are trained using randomly selected methods. Therefore, RF has high diversity and accuracy [13]. XGBoost is a gradient-boosting algorithm based on decision tree models, suitable for classification and regression problems. Gradient boosting is an iterative algorithm that calculates the error by analyzing the loss function of the data at each iteration, then uses a gradient descent algorithm to update the parameters of the decision tree model, making the model better at offsetting errors in the next iteration. This process is repeated multiple times, with each iteration adding a decision tree model, and all decision tree models are combined into an ensemble model [14]. The schematic of machine learning model construction is shown in Fig. (**1**).

During the training of the machine learning model, in addition to the parameters of the model, there are also some hyperparameters that need to be determined, which can have a significant impact on the performance of the model. The grid search algorithm enumerates all possible combinations of hyperparameters and trains and evaluates the model on each combination to find the best-performing hyperparameter combination for model construction [15].

#### 2.2.3 Evaluation of Machine Learning Models

In model evaluation, the following methods are used: (1) confusion matrix of the test set, which is used to evaluate the prediction performance of classification models. Each row represents the true class, and each column represents the predicted class of the model. (2) ROC curve of the test set, which is a graphical representation method for evaluating the performance of classification models. Each point on the graph represents the performance of the model at different discrimination thresholds. The coordinate system of the ROC curve consists of two axes, namely the true positive rate (TPR) and the false positive rate (FPR). As the discrimination threshold of the classification model changes from high to low, the change in TPR and FPR will form a curve. An ideal ROC curve should be as close to the upper left corner as possible, *i.e*., the difference between TPR and FPR should be as large as possible [8]. (3) multiple indicators of the test set, including accuracy, represent the ratio of correctly predicted data to the total data; precision represents the proportion of truly positive data in the data predicted as positive by the classification model; recall represents the proportion of truly positive data predicted by the classification model to the actual positive data; F1 score is the harmonic mean of precision and recall, combining the two indicators to represent the prediction effect of the model; AUC: represents the area under the ROC curve, which is an indicator for evaluating the performance of classification models. The greater the value, the more accurate the prediction; Matthew’s correlation coefficient (MCC): a binary evaluation indicator used to evaluate the accuracy of classification models. It considers both the accuracy and completeness of the classification model. The value of MCC is between -1 and 1. The closer the value is to 1, the better the performance of the classification model [16]; Kappa: a binary evaluation indicator used to evaluate the accuracy of classification models and the correlation between the accuracy of random predictions. The value of Kappa is between 0 and 1. The closer the value is to 1, the better the performance of the classification model [17]. Brier score: is an indicator for evaluating the quality of prediction results, used to evaluate the accuracy of prediction results. The smaller the value of the Brier score, the more accurate the prediction result [18]. (4) Accuracy, F1 score, and ROC curve of 10-fold cross-validation. 10-fold cross-validation divides the training data into ten parts. Each time, one part is selected as the validation data, and the remaining nine parts are used as the training data for model training. The entire process is repeated ten times. Finally, the average of each validation result is taken as the final validation result of the model. 10-fold cross-validation can evaluate the generalization ability of the model and avoid overfitting problems.

### 2.3 Deep Learning

A molecular graph is a method of representing molecules through graphics, where atoms and bonds of the molecule are represented as nodes and edges in the graph. For each target dataset, DeepChem [19] generates molecular graphs based on SMILES, which are then used to construct a Graph Convolutional Network (GCN). GCN is a special type of Convolutional Neural Network used for processing graphical data such as molecular structures. The difference between graph convolution and normal convolution is that graph convolution operates on the graph structure rather than the grid structure [20]. The working principle of GCN is to extract features from adjacent information of the graphical structure by employing a specific graph convolution operation and to continuously combine these features through multiple layers of convolution operations to generate a representation for the graph. The core part of the graph convolution operation is a graph convolution kernel, which is used to compute the weighted sum between the current node and its adjacent nodes. The pooling layer after the convolution layer is a downsampling technique that reduces the amount of data by taking the maximum value within a local region. It is typically used to reduce the spatial dimension of the graph convolution features, decrease the number of parameters, and simplify calculations. The fully connected layer is a neural network layer that is fully connected and adds a nonlinear transformation to the graph convolution features. It is usually used to calculate the final classification results [21]. In the model construction process, the Bayesian optimization algorithm is used to optimize hyperparameters such as learning rate, batch size, epoch, and regularization coefficient. The Bayesian optimization algorithm makes prior distribution assumptions for the parameters to be optimized using probability models and updates the model parameters based on the data to achieve the goal of the optimal parameters [22]. After training the model with the optimal hyperparameters, the model performance is evaluated using the test set. The schematic of deep learning model construction is shown in Fig. (**2**).

### 2.4 Integrated Model

Based on the model evaluation results, two machine learning models and the Graph Convolutional Neural Network model are selected to construct an integrated model for each target. The construction method of the integrated model is a soft ensemble, which aims to combine the prediction results of multiple models to generate the final prediction result [23]. By averaging the weighted predictions of different models, the advantages of each model can be combined to reduce errors caused by using individual models alone, *i.e*., to improve the stability and accuracy of the model and reduce overfitting of the model. After constructing the integrated model using the optimal weight combination, the model performance is evaluated in the same way as the GCN.

### 2.5 Identification of Chemical Components in Xanthocerais lignum based on HPLC-Q-Exactive-MS

Instruments used include Thermo Scientific UltiMate 3000 High-Performance Liquid Chromatography System, Q Exactive ^TM^hybrid quadrupole-Orbitrap mass spectrometer, high-speed centrifuge, rotary evaporator system, analytical balance and electronic balance. Reagents used include chromatographic methanol, chromatographic acetonitrile, chromatographic acetic acid, analytical grade anhydrous ethanol and ultrapure water. The herbal medicine used is Xanthocerais lignum (dried stem or branch of the *Xanthoceras sorbifolia* Bunge. tree in the Sapindaceae family).

#### 2.5.1 Preparation of the Sample Solution

Crush the Xanthocerais lignum herb slices and weigh out 800 g of crude powder. Extract three times with 70% ethanol reflux, each time for 2 hours, filter, combine the filtrate, and recover it until there is no alcohol taste, yielding 112.5 g of extract. Precisely weigh 0.2532 g of the ethanol extract and dissolve it in chromatographic methanol. Add it to a 25 mL volumetric flask and centrifuge it for 15 minutes at 12000 r. min^-1^ before analyzing the supernatant. In the previous experiment, we investigated the influence of different extracting solvents (water, 50% methanol, 70% methanol, methanol, 50% ethanol, 70% ethanol, ethanol) on the content of six flavonoid compounds (catechin, epicatechin, (-)-epigallocatechin, myricetin, dihydromyricetin, dihydroquercetin) in Xanthocerais lignum. The results revealed that the highest content of each component was observed when 70% ethanol was used as the extracting solvent.

#### 2.5.2 Chromatographic Conditions

Chromatographic column: Symmetry^®^ C_18_ column (250 mm × 4.6 mm, 5.0 μm); mobile phase: acetonitrile (A) ~ 0.4% acetic acid water (B); elution gradient: 0 ~ 5 min, 5% ~ 10% A ; 5 ~ 15 min, 10% ~ 12% A; 15 ~ 40 min, 12% ~ 20% A; 40 ~ 50 min, 20% ~ 30% A; 50 ~ 55 min, 30% ~ 40% A; 55 ~ 70 min, 40% ~ 100% A; 70 ~ 75 min, 100% A; 75 ~ 90 min, 100% ~ 5% A; 90 ~ 100 min, 5% A; injection volume: 10µL; column temperature: 20 °C; flow rate: 1 mL.min^-1^.

#### 2.5.3 Mass Spectrometry Conditions

Use the ESI source positive/negative ion mode detection for mass spectrometry. The detection parameters are as follows: ion source voltage of 4 kV (+)/3.2 kV (-); sheath gas volume flow rate of 40 L.min^-1^ (+)/35 L.min^-1^ (-); fragmentation voltage of 300 V; drying gas temperature of 350 °C; saturated auxiliary gas volume flow rate of 2 L.min^-1^; spray air pressure of 45 psig; high purity nitrogen gas is used as atomizing gas; data acquisition range is 100 ~ 1100 m/z, using full MS-ddMS2 scanning method.

#### 2.5.4 Identification of Chemical Components

HPLC-Q-Exactive-MS technology is used to qualitatively analyze the chemical components in Xanthocerais lignum ethanol extract. First, a chemical composition information table for Xanthocerais lignum was constructed based on existing literature reports. Then, based on the relative molecular weight of each chromatographic peak measured in reality and the accurate relative molecular weight provided by theory, the molecular formula corresponding to each chromatographic peak is preliminarily identified. Search the constructed chemical information table based on the molecular formula to find the target compound that matches. Combine the primary and secondary mass spectrometry fragmentation data of the target peak and compare it with the relevant data in the literature, using the mass spectrometry fragmentation rules for this type of chemical composition provided in the literature to identify their chemical structures. For the chromatographic peaks that cannot be matched, Compound Discoverer 3.2 and MassBank (www.massbank.jp) databases are used for primary and secondary mass spectrometry information searching and matching.

### 2.6 Prediction of Xanthocerais lignum's Anti-RA Active Ingredients

Based on LC-MS identification of compounds present in Xanthocerais lignum, supplemented by a search through relevant literature, a compound library is constructed. The SMILES for each compound are obtained from PubChem (pubchem.ncbi.nlm.nih.gov). After characterizing and generating molecular graphs for each compound using the same method as the respective datasets, an integrated model is used for each target to predict the activity of the chemical components in Xanthocerais lignum. Compounds with predicted probabilities greater than 0.5 are screened as potentially effective anti-RA active ingredients present in Xanthocerais lignum.

## 3 RESULTS AND DISCUSSION

### 3.1 Dimensionality Reduction Analysis of Molecular Characteristics

For each target dataset, 208 molecular descriptors, 1024 Morgan fingerprints, and 167 MACCS keys, totaling 1399 molecular features, were generated through RDkit to characterize the molecules. Principal component analysis (PCA) was performed on the 208 molecular descriptors to analyze the distribution of active and inactive molecules in a two-dimensional space. As shown in Fig. (**3**), active and inactive molecules from the seven target datasets were mainly distributed in the range of PC1 (-2 to 3) and PC2 (-1.5 to 2). Except for the PI3K dataset, which exhibited a large overlap in the distribution of active and inactive molecules, the other target datasets showed significant differences in the distribution of active and inactive molecules. Molecular descriptors mainly reflect the structure and properties of molecules, indicating that there are significant differences in the structure and properties of active and inactive molecules for each target.

PCA is a linear dimensionality reduction method and may not fit well when dealing with nonlinear features. Therefore, the nonlinear dimensionality reduction method t-distributed stochastic neighbor embedding (t-SNE) was used to reduce the 1024 Morgan fingerprints and 167 MACCS keys, totaling 1191 molecular fingerprints, to analyze the distribution of active and inactive molecules in a two-dimensional space. As shown in Fig. (**4**), the PI3K, AKT, and EGFR datasets were mainly distributed in the range of t-SNE1 and t-SNE2 (-100 to 100), while the IGF1R and IRAK4 datasets were mainly distributed in the range of t-SNE1 and t-SNE2 (-80 to 80), and the Bcl2 and FAK datasets were mainly distributed in the range of t-SNE1 (-60 to 70) and t-SNE2 (-70 to 70), respectively. Molecular fingerprints reflect the structural characteristics of molecules, and the visualization results show that active and inactive molecules in each target dataset have significant differences in structure, consistent with the results of PCA analysis. Machine learning models can learn these differential features and accurately classify active and inactive molecules. In addition, the visualization results show that each target dataset has chemical diversity in molecular structure, and molecules with diverse structures can improve the generalization ability of machine learning models and enhance model performance.

To verify the rationality of the division of each target dataset, t-SNE analysis was performed on the training set and test set to observe their distribution in a three-dimensional space. As shown in Fig. (**5**), the distribution of the training and test sets of each target in space is basically consistent, indicating that the molecules in the training and test sets have similarities in structure and properties, and the division of each target dataset is reasonable.

### 3.2 Results of Hyperparameter Optimization for Models

In machine learning and deep learning, hyperparameters refer to those parameters that need to be manually set by humans rather than model parameters learned automatically from training data. Different models have different hyperparameters that need to be set. In LR, the penalty is the type of regularization term, including L1 regularization and L2 regularization, while C is the regularization hyperparameter that controls the strength of regularization. In KNN, the neighbors parameter represents the number of neighbors selected, which is the number of k nearest neighbors used to determine the classification label, and the weights parameter is used to determine the weights of the neighbors. In SVM, the kernel parameter is used to select the kernel function, which is used to map the data from the original space to a higher-dimensional feature space for better data separation. In RF, the estimator parameter represents the number of decision trees used to construct the random forest, and the max depth parameter is used to control the maximum depth of decision trees. In contrast, XGBoost requires setting the learning rate parameter in addition to the estimator parameter to control the weight of each weak learner in the gradient-boosting process. In GCN, four main hyperparameters are set, namely learning rate, batch size, epoch, and L2 regularization, which control the step size of each parameter update in the model, the number of samples used in each training, the iteration times of the model, and the complexity of the model, respectively.

The optimal hyperparameters for each model are shown in Table **1**. It can be seen that some models do not need too much adjustment of hyperparameters to achieve good model performance, while others need multiple adjustments according to different datasets. This may be due to the fact that different models have different complexity and bias/variance characteristics. Models with lower complexity usually have weaker fitting ability to data, such as LR and KNN models, and hyperparameters have little influence on model performance, so they do not need too much adjustment. On the other hand, models with more complex structures can better fit data but also require more hyperparameter adjustments, such as XGBoost and GCN models. In addition, the size of the dataset also affects the difficulty of hyperparameter adjustment. For smaller datasets, overfitting is more likely to occur, which means that smaller hyperparameter values and stronger regularization are needed to control the model's complexity. For larger datasets, models usually need higher complexity to fully utilize the information in the data.

### 3.3 Model Evaluation

#### 3.3.1 Machine Learning Model Evaluation and Comparison

The various metrics on the test set can reflect the model's performance to some extent comprehensively. As shown in Table **2**, different models perform differently on the same target dataset, and the same model may perform differently on different target datasets. Therefore, it is necessary to select models with good performance based on the evaluation results and integrate them to achieve better overall performance. SVM and XGBoost outperform other models in terms of precision, F1 score, and AUC on the PI3K, AKT, EGFR, and IRAK4 target datasets. Although RF has high recall rates on the PI3K and AKT datasets, its precision and accuracy are relatively low. LR and KNN have certain gaps compared to SVM and XGBoost on all metrics. On the Bcl2 and IGF1R target datasets, the performance of SVM, RF, and XGBoost models is similar, and further determination is required through 10-fold cross-validation. On the FAK target dataset, RF and XGBoost outperform other models on all metrics.

Evaluating the model performance solely based on the test set may not reflect the model's generalization ability. Therefore, 10-fold cross-validation is used to compare the accuracy and F1 score of different models on different target datasets, and the results are visualized using boxplots. The median value is more representative of the general level than the mean value, which is susceptible to outlier influence. As shown in Fig. (**6**), SVM and XGBoost models outperform other models in terms of accuracy and F1 score on the PI3K, AKT, and EGFR target datasets. The results are consistent with the test set results. For the IRAK4 target dataset, the performance of the SVM model is better than that of other models in terms of accuracy and F1 score, but its performance on the test set is not as good as that of the XGBoost model, which may be due to the unstable evaluation results caused by different data splitting methods. On the Bcl2 and IGF1R target datasets, the 10-fold cross-validation results of all models are excellent, indicating that these models have strong generalization ability on these two datasets and can adapt well to new data to achieve good prediction results. XGBoost performs best on the FAK target dataset, and the accuracy of RF is slightly lower than that of SVM, but its F1 score is higher. F1 score comprehensively evaluates a model's precision and recall, which is less susceptible to the influence of sample imbalance than accuracy, so the performance of the RF model on the FAK target dataset may be slightly better than that of the SVM model.

Furthermore, the performance of each model is evaluated using the ROC curve through 10-fold cross-validation, and the closer the average AUC value is to 1, the better the model's performance. ROC curves of different machine learning models in 10-fold cross-validation are shown in Fig. (**7**). Based on the AUC values, the best model is selected for each target dataset. The selected models have AUC values greater than 0.92 on the PI3K, AKT, Bcl2, EGFR, FAK, and IRAK4 target datasets, indicating that they can classify unknown compounds well. Finally, based on the test set results and 10-fold cross-validation results, the models with good performance are selected for each target dataset to build an integrated model. SVM and XGBoost perform best on the PI3K, AKT, EGFR, and IRAK4 datasets, while RF and XGBoost perform best on the Bcl2 and FAK datasets, and RF and SVM perform best on the IGF1R dataset.

#### 3.3.2 Evaluation of Deep Learning Models

GCN is a commonly used deep learning model that exhibits excellent performance when processing graph data. Through multiple layers of graph convolution operations, GCN can gradually learn the feature representation of molecular graph nodes, thereby accurately classifying unknown compounds. As shown in Table **2**, the precision of GCN is higher than that of machine learning models on various target datasets, indicating that it can accurately judge active molecules and is less likely to misjudge inactive molecules. However, precision cannot reflect the coverage of the model for all active molecules, and usually, precision and recall are mutually restrictive. Machine learning models have lower accuracy than GCN models but higher recall and AUC scores. Therefore, in this study, we intend to establish an integrated model to combine the advantages of various models and improve the model's generalization ability and stability.

### 3.4 Construction and Evaluation of Integrated Model

The integrated model can combine the advantages of multiple basic models and has better model performance than a single model. Based on the evaluation results of the above models, we selected two machine learning models with better performance and GCN models for each target dataset to integrate. By traversing various model weight combinations, we determined the best model weight, and the results are as follows: PI3K: [GCN: SVM: XGBoost = 0.3: 0.4: 0.^3^]; AKT: [GCN: SVM: XGBoost = 0.3: 0.3: 0.^4^]; Bcl2: [GCN: RF: XGBoost = 0.2: 0.4: 0.^4^]; EGFR: [GCN: SVM: XGBoost = 0.2: 0.3: 0.^5^]; IGF1R: [GCN: RF: SVM = 0.3: 0.4: 0.^3^]; FAK: [GCN: RF: XGBoost = 0.4: 0.2: 0.^4^]; IRAK4: [GCN: SVM: XGBoost = 0.3: 0.3: 0.^4^].

Comparing the performance of GCN models and integrated models through the confusion matrix, where the horizontal axis represents the predicted label, the vertical axis represents the true label, and the four indicators are true negative, false positive, false negative, and true positive. As shown in Fig. (**8**), the integrated model makes up for the low recall of the GCN model while having higher accuracy. The performance of the integrated model was evaluated using the test set, and as shown in Table **2**, the integrated model has higher accuracy and precision than other machine learning models. Moreover, the AUC of the integrated model on all target datasets is greater than 0.94, and the MCC, Kappa, and Brier scores are also better than other models. In summary, the establishment of an integrated model through soft integration is feasible. The stability and accuracy of the integrated model have been greatly improved compared to a single model, and it can better predict the activity of unknown compounds.

### 3.5 Analysis Results of HPLC-Q-Exactive-MS

The sample was detected using negative ion mode, and a good chromatographic separation and signal intensity were observed in the total ions current (TIC) chart. LC-MS analysis results showed that a total of 37 compounds were identified from the ethanol extract of Xanthocerais lignum, mainly flavonoids. The TIC chart of the ethanol extract of Xanthocerais lignum under negative ion mode is shown in Fig. (**9**), and detailed information on the identified chemical components is shown in Table **3**. Based on the component identification information and the TIC chart, it can be seen that (-)-epigallocatechin, epicatechin, dihydromyricetin and myricetin have relatively high relative contents in Xanthocerais lignum, with retention times of 13.05 min, 22.87 min, 24.12 min, and 48.41 min, respectively.

### 3.6 Chemical Components of Xanthocerais Lignum

In the above study, we identified 37 compounds in Xanthocerais lignum using HPLC-Q-Exactive-MS and supplemented them with relevant literature to construct a compound library of Xanthocerais lignum, which includes a total of 69 compounds, including 27 flavonoids, 5 triterpenoids, 2 phenylpropanoids, 3 steroids, 8 phenols, 4 quinones, 10 organic acid compounds and 10 other compounds. The supplementary chemical composition of Xanthocerais lignum is shown in Table **4**. As the main chemical component of Xanthocerais lignum, flavonoids can reduce inflammation by activating the antioxidant pathway, inhibiting cyclooxygenase, and regulating the expression of cytokines [31], and maybe the main active component of Xanthocerais lignum in exerting its anti-RA effect.

### 3.7 Integrated Model Prediction Results

The integrated models with different targets were used to predict and screen the Xanthocerais lignum compound library. The predicted results can be found in Table **5**, and the structures of active ingredients can be seen in Fig. (**10**). Among them, 27 active ingredients targeting PI3K were predicted, and it can be observed that most of these compounds are flavonoids, which typically contain multiple hydroxyl groups that can form hydrogen bonds with PI3K targets, thereby inhibiting their activity. Additionally, many of these compounds contain oxygen atoms at the C-3 and C-4 positions, which can interact with the subunits of the PI3K protein, thereby altering its conformation and inhibiting its activity. Moreover, many of these compounds have been confirmed to have PI3K inhibitory activity, such as myricetin, which can competitively bind to the ATP binding site of the PI3K enzyme, thereby blocking its catalytic activity and affecting the activation state of downstream Akt [41]. Three active ingredients targeting AKT were predicted, all of which are flavonoids. Cinnamtannin B1 and procyanidin A-2 have similar skeletal structures consisting of anisotropic units that have good electron density and can form stable π-π stacking structures, thereby interacting with the aromatic amino acids on the protein surface and possibly playing a critical role in the activation and inhibition process of the AKT target. Three active ingredients targeting Bcl2 were predicted. They all contain a cyclic lipid structure and multiple hydroxyl groups. The cyclic lipid structure can provide spatial conformation for the molecule, while the hydroxyl groups can interact with the amino acid residues through hydrogen bonding, ultimately affecting the positioning and activity of the molecule on the Bcl2 target. Twelve active ingredients targeting EGFR were predicted, all of which are flavonoids. The two compounds with the highest activity are myricetin and its 3-*O*-rhamnoside, and it is speculated that compounds with this type of structure can bind well to the EGFR target and exert an inhibitory effect.

Six active ingredients targeting IGF1R were predicted, all of which contain a benzene ring or a benzopyran skeleton, and most of them contain hydroxyl groups. These structural features may be related to their inhibitory effect on the IGF1R target, as hydroxyl groups can form hydrogen bonds or hydrophobic interactions with the amino acid residues in the ligand binding site of IGF1R. In addition, the benzene ring or benzopyran skeleton can form π-π stacking with the aromatic ring in the ligand binding site of IGF1R [42]. Three active ingredients targeting FAK and two active ingredients targeting IRAK4 were predicted, and it can be observed that they all have one or more sugar substitutions. These sugar substitutions can increase the bioavailability of the compound and may have an important impact on the affinity of the compound with the target.

By performing frequency statistics on the integrated model prediction results, it can be found that isorhamnetin-3-*O*-glucoside, myricetin, rutinum, and cinnamtannin B1 have inhibitory effects on three or more targets, while dihydromyricetin, myricitrin, daucosterol, *etc*., have inhibitory effects on two targets. Moreover, myricetin and dihydromyricetin have relatively high relative contents in Xanthocerais lignum, so these components may be the main active ingredients for its anti-RA effect.

The numbers in parentheses in Table **5** correspond to the compound structure in Fig. (**10**).

## CONCLUSION

This study successfully applied LC-MS and AI techniques to predict the anti-RA active components in Xanthocerais lignum. We used various machine learning algorithms and GCN to generate an integrated model and evaluated its performance by comparing the confusion matrices and relevant evaluation metrics on the test sets. The results showed that the integrated model had better predictive performance than individual models and exhibited higher accuracy and precision on all target data sets. Additionally, we conducted a dimensionality reduction analysis of molecular features to better understand the differences between active and inactive molecules. The results showed that active and inactive molecules in each target data set had significant differences in structure and properties. The integrated model can learn these differential features to accurately classify active and inactive molecules and predict the anti-RA active components in Xanthocerais lignum.

Through activity screening of compounds in Xanthocerais lignum, we identified several compounds with potential anti-RA activity, such as isorhamnetin-3-*O*-glucoside, myricetin, rutinum, cinnamtannin B1, dihydromyricetin, *etc*. These results provide a valuable reference for further research on the anti-RA active ingredients in Xanthocerais lignum. However, this study also has some limitations, such as possible errors or missing data in the data preparation process, and our model may be affected by factors such as dataset size and molecular descriptor selection. Therefore, future improvements in this method are needed to enhance predictive accuracy and reliability.

In summary, this study successfully applied LC-MS and AI techniques to predict the anti-RA active components in Xanthocerais lignum and provided valuable references for further research on the pharmacology and active natural products of Xanthocerais lignum. Our research results indicate that LC-MS and AI techniques have broad application prospects in natural product pharmacology and structure-activity relationship studies. In the future, we will further explore the application of these techniques in other natural products and continuously improve the method to provide more powerful support for the discovery of new active molecules in medicinal plants.

## AUTHORS’ CONTRIBUTIONS

All authors have read and approved the final manuscript for publication and take full responsibility for the accuracy and integrity of the work.

## Figures and Tables

**Fig. (1):**
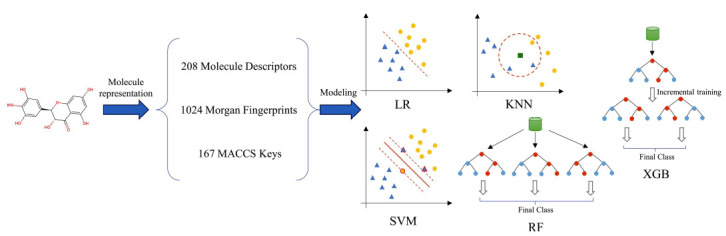
Construction of machine learning model.

**Fig. (2):**
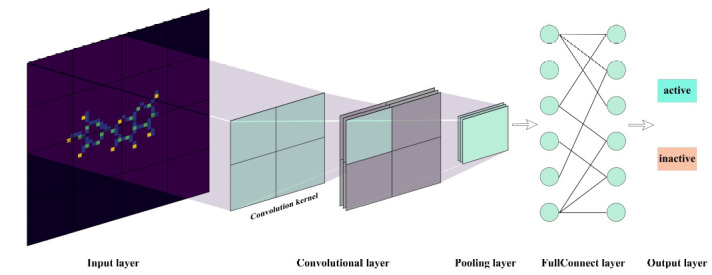
Construction of deep learning model.

**Fig. (3):**
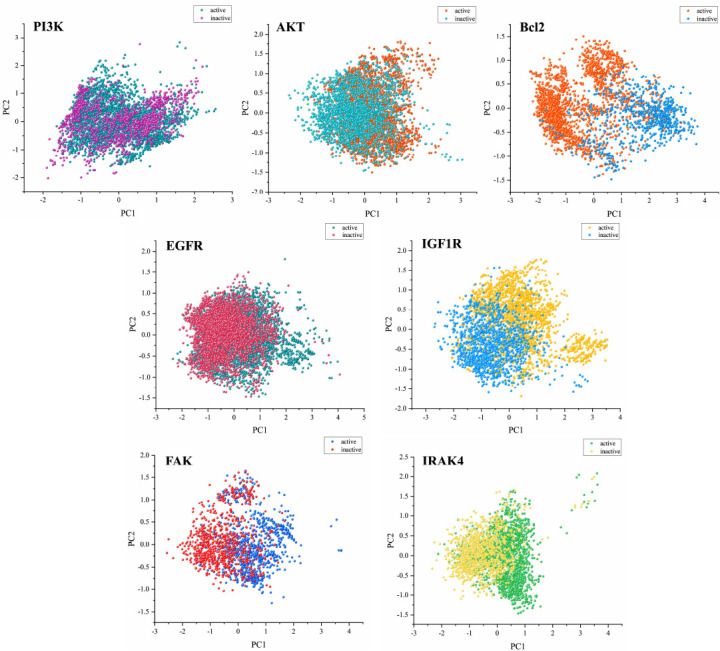
PCA Analysis results of active and non-active molecules in each target dataset.

**Fig. (4):**
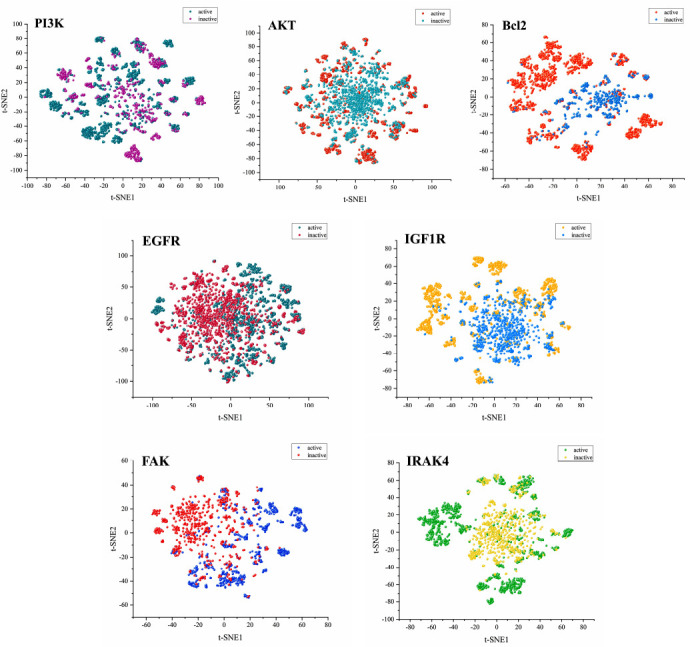
t-SNE analysis results of active and non-active molecules in each target dataset.

**Fig. (5):**
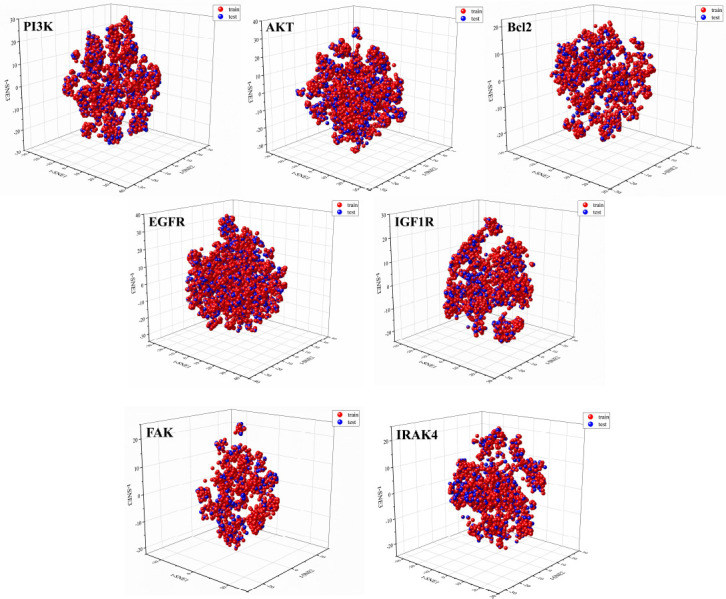
t-SNE analysis results of training and testing molecules in each target dataset.

**Fig. (6):**
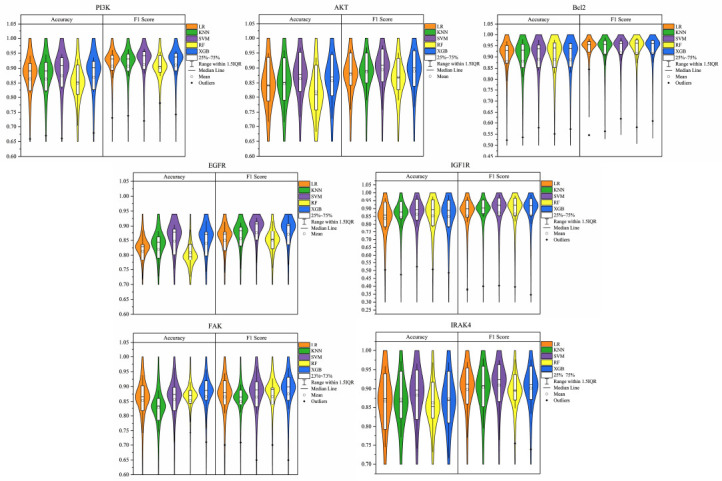
Violin plots of accuracy and F1 scores of different machine learning models in 10-fold cross-validation.

**Fig. (7):**
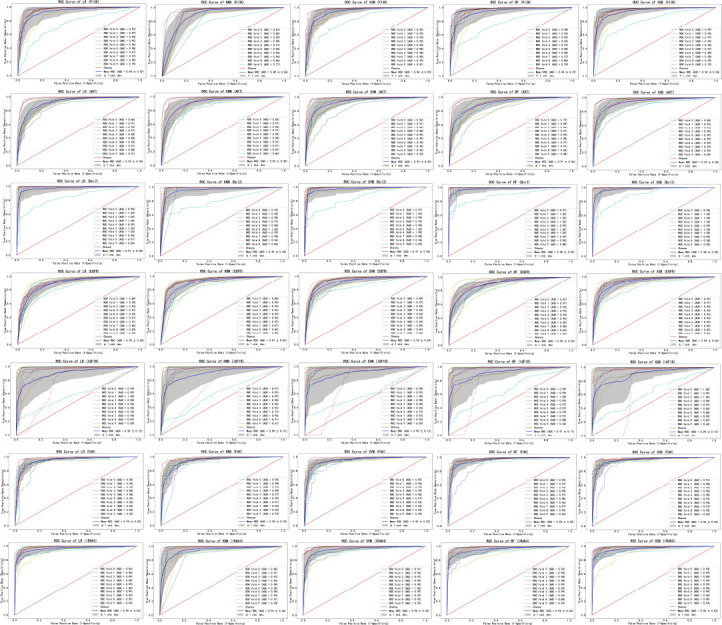
ROC curves of different machine learning models in 10-fold cross-validation.

**Fig. (8):**
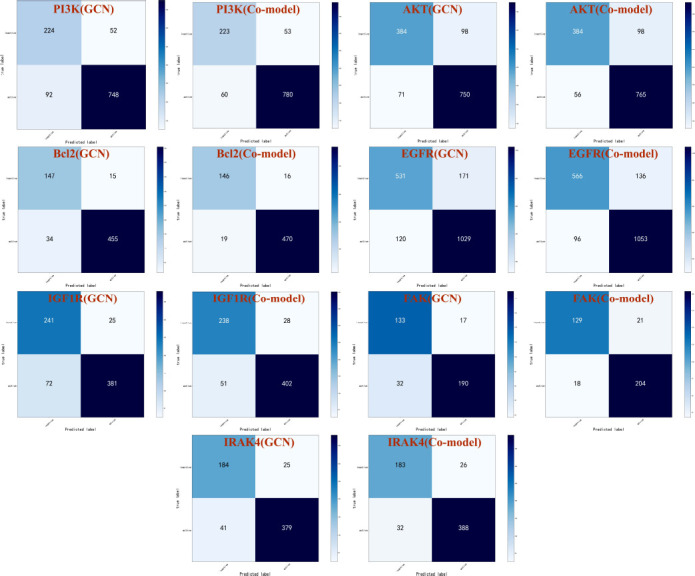
Confusion matrices of GCN and co-model in each target dataset.

**Fig. (9):**
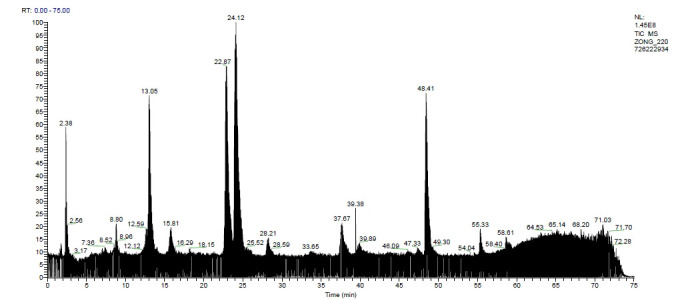
TIC of HPLC-Q-Exactive-MS analysis of xanthocerais lignum.

**Fig. (10):**
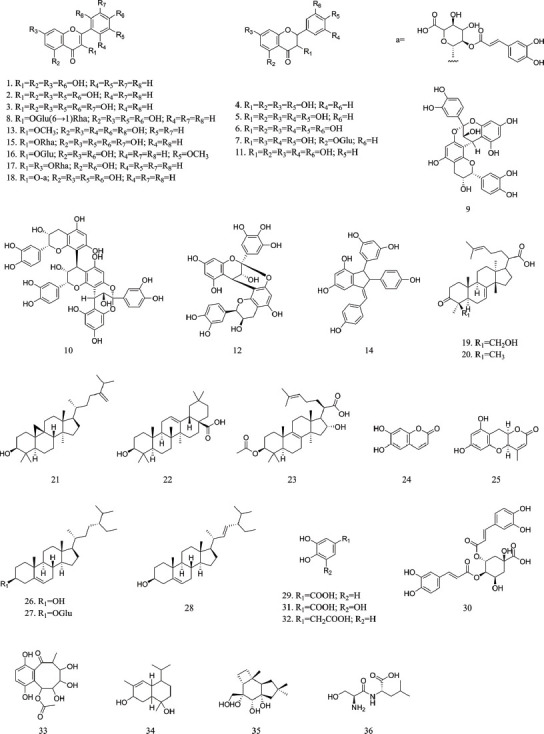
Anti-RA activity natural products in Xanthocerais lignum.

**Table 1:** Results of hyperparameter optimization for each model.

**Model**	**Hyperparameter**	**PI3K**	**AKT**	**Bcl2**	**EGFR**	**IGF1R**	**FAK**	**IRAK4**
LR	penalty	L2	L2	L2	L2	L2	L2	L2
	C	0.1	0.1	0.1	0.1	0.1	1	0.1
KNN	neighbors	3	18	10	7	7	16	6
	weights	uniform	distance	distance	distance	distance	distance	distance
SVM	kernel	rbf	rbf	rbf	rbf	rbf	rbf	rbf
	C	10	10	10	10	1	10	10
RF	estimators	300	500	200	300	500	200	500
	max depth	10	10	10	10	10	10	10
XGBoost	learning rate	0.1	0.1	0.01	0.1	0.01	0.1	0.1
	estimators	500	400	400	200	400	250	300
	max depth	9	8	6	8	6	8	5
GCN	learning rate	0.000385	0.000105	0.00130	0.00263	0.00352	9.08	0.000933
	batch size	8	16	8	16	32	8	8
	epoch	100	100	100	100	100	100	100
	l2 reg	0	1	0.1	0.1	0.1	1	0.1

**Table 2:** Results of testing sets for each target dataset.

**Target**	**Model**	**Accuracy**	**Precision**	**Recall**	**F1**	**AUC**	**MCC**	**Kappa**	**BS**
PI3K	LR	0.874	0.901	0.935	0.918	0.935	0.649	0.647	0.126
	KNN	0.884	0.925	0.921	0.923	0.919	0.691	0.691	0.116
	SVM	0.896	0.932	0.930	0.931	0.943	0.721	0.721	0.104
	RF	0.867	0.868	0.971	0.917	0.934	0.618	0.594	0.133
	XGB	0.883	0.921	0.924	0.922	0.943	0.684	0.684	0.117
	GCN	0.871	0.935	0.890	0.912	0.931	0.672	0.669	0.129
	Co-model	0.899	0.936	0.929	0.932	0.949	0.730	0.730	0.101
AKT	LR	0.851	0.847	0.932	0.887	0.917	0.676	0.669	0.149
	KNN	0.872	0.860	0.951	0.903	0.928	0.722	0.714	0.128
	SVM	0.878	0.878	0.937	0.906	0.934	0.735	0.732	0.122
	RF	0.837	0.816	0.959	0.881	0.926	0.649	0.628	0.163
	XGB	0.876	0.876	0.935	0.905	0.943	0.730	0.727	0.124
	GCN	0.870	0.884	0.914	0.899	0.930	0.719	0.719	0.130
	Co-model	0.882	0.886	0.932	0.909	0.946	0.744	0.742	0.118
Bcl2	LR	0.935	0.957	0.957	0.957	0.980	0.827	0.827	0.065
	KNN	0.931	0.951	0.957	0.954	0.974	0.814	0.814	0.069
	SVM	0.942	0.961	0.961	0.961	0.974	0.844	0.844	0.058
	RF	0.939	0.965	0.953	0.959	0.986	0.838	0.838	0.061
	XGB	0.946	0.963	0.965	0.964	0.987	0.856	0.856	0.054
	GCN	0.925	0.968	0.930	0.949	0.977	0.809	0.806	0.075
	Co-model	0.946	0.967	0.961	0.964	0.986	0.857	0.857	0.054
EGFR	LR	0.834	0.848	0.892	0.869	0.907	0.642	0.640	0.166
	KNN	0.857	0.863	0.915	0.888	0.926	0.692	0.690	0.143
	SVM	0.874	0.892	0.907	0.899	0.932	0.731	0.731	0.126
	RF	0.829	0.828	0.914	0.869	0.916	0.630	0.624	0.171
	XGB	0.867	0.878	0.911	0.894	0.939	0.714	0.713	0.133
	GCN	0.843	0.858	0.896	0.876	0.913	0.662	0.661	0.157
	Co-model	0.875	0.886	0.916	0.901	0.942	0.732	0.731	0.125
IGF1R	LR	0.875	0.904	0.896	0.900	0.933	0.732	0.732	0.125
	KNN	0.876	0.899	0.905	0.902	0.973	0.734	0.734	0.124
	SVM	0.889	0.935	0.885	0.909	0.934	0.768	0.766	0.111
	RF	0.878	0.910	0.894	0.902	0.940	0.739	0.739	0.122
	XGB	0.873	0.902	0.896	0.899	0.935	0.729	0.729	0.127
	GCN	0.865	0.938	0.841	0.887	0.929	0.727	0.721	0.135
	Co-model	0.890	0.935	0.887	0.911	0.944	0.770	0.768	0.110
FAK	LR	0.858	0.872	0.892	0.882	0.930	0.703	0.702	0.142
	KNN	0.844	0.839	0.914	0.875	0.925	0.673	0.669	0.156
	SVM	0.863	0.880	0.892	0.886	0.940	0.714	0.714	0.137
	RF	0.871	0.882	0.905	0.893	0.931	0.731	0.730	0.129
	XGB	0.884	0.894	0.914	0.904	0.943	0.759	0.759	0.116
	GCN	0.868	0.918	0.856	0.886	0.931	0.733	0.731	0.132
	Co-model	0.895	0.907	0.919	0.913	0.944	0.782	0.781	0.105
IRAK4	LR	0.890	0.907	0.931	0.919	0.953	0.750	0.749	0.110
	KNN	0.887	0.911	0.921	0.916	0.941	0.744	0.744	0.113
	SVM	0.898	0.926	0.921	0.924	0.958	0.771	0.771	0.102
	RF	0.889	0.913	0.921	0.917	0.958	0.748	0.748	0.111
	XGB	0.901	0.934	0.917	0.925	0.964	0.780	0.780	0.099
	GCN	0.895	0.938	0.902	0.920	0.946	0.769	0.768	0.105
	Co-model	0.908	0.937	0.924	0.930	0.965	0.794	0.794	0.092

**Table 3:** Chemical composition identification of Xanthocerais lignum by HPLC-Q-Exactive-MS.

**No.**	**RT (min)**	**Chemical Formula**	**Measured Value (m/z)**	**Theoretical Value (m/z)**	**Fragmentation**	**Compounds**	**References**
1.	2.22	C_6_H_12_O_6_	179.05486[M-H]^-^	179.05611	161.04422,101.02271,97.02795,89.02294,87.00726,71.01231,59.01013	D-glucose	-
2.	2.29	C_6_H_14_O_6_	181.07047[M-H]^-^	181.07176	163.05971,119.03349,101.02290,89.02283,71.01228,59.01228	dulcitol	-
3.	2.32	C_5_H_12_O_5_	151.05983[M-H]^-^	151.06120	101.02297,89.02290,71.01234,59.01234	D-(+)-arabitol	-
4.	2.41	C_9_H_18_N_2_O_4_	217.00098[M-H]^-^	217.11938	174.99092,132.05016,86.12391	serylleucine	-
5.	2.47	C_12_H_22_O_11_	341.10773[M-H]^-^	341.10893	179.05479,161.04387,119.03344,113.02285,101.02293,89.02284,71.01227,59.01230	α,α-trehalose	-
6.	5.55	C_29_H_50_O	413.16617[M-H]^-^	413.37888	397.27887,353.14572,207.08623,158.98982,101.02293,59.01233	*β*-sitosterol	[24]
7.	5.97	C_7_H_6_O_5_	169.01312[M-H]^-^	169.01425	125.02294	gallic acid	[25]
8.	7.40	C_32_H_50_O_5_	513.13757[M-H]^-^	513.35854	397.26346,159.08020,91.75667,79.30959	3-*O*-acetyl-16alpha-hydroxytrametenolic acid	-
9.	7.50	C_22_H_22_O_12_	477.16089[M-H]^-^	477.10385	299.00562,284.99008,242.96591,198.97444	isorhamnetin-3-*O*-glucoside	-
10.	10.02	C_7_H_6_O_4_	153.01808[M-H]^-^	153.01933	109.02802	protocatechuic acid	[24, 25]
11.	13.05	C_15_H_14_O_7_	305.06567[M-H]^-^	305.06668	261.07675,219.06531,179.03368,167.03358,139.03859,137.02303,125.02293	(-)-epigallocatechin	[24, 26]
12.	15.81	C_15_H_14_O_6_	289.07156[M-H]^-^	289.07176	245.08133,206.05318,203.07050,125.02300,109.02809	catechin	[25, 27]
13.	18.16	C_9_H_6_O_4_	177.01816[M-H]^-^	177.01933	133.02818,105.03335	esculetin/6,7-dihydroxycoumarin	[24]
14.	19.28	C_27_H_30_O_14_	577.13550[M-H]^-^	577.15628	407.07693,289.07166,245.08171,161.02321,137.02295,125.02300	kaempferitrin	-
15.	21.20	C_21_H_24_O_11_	451.13779[M-H]^-^	451.12458	397.25290,320.31161,274.60251,229.94392,212.84039,176.54782,159.06668,153.77745	epicatechin-5-*O*-*β*-D-glucopyranaoside	[24, 28]
16.	22.98	C_15_H_14_O_6_	289.07089[M-H]^-^	289.07176	245.08134,206.05290,203.07045,179.03381,151.03880,137.02309,125.02300,109.02808,97.02811	epicatechin	[24, 25, 29]
17.	23.05	C_6_H_10_O_7_	193.01312[M-H]^-^	193.03537	165.01799,137.02293,121.02817	glucuronic acid	-
18.	24.02	C_30_H_24_O_16_	639.09863[M-H]^-^	639.09916	301.03491,257.04495,215.03409,193.01318,175.00232,125.02290	quercetin-3-(2''-caffeylglucuronide)	-
19.	24.20	C_15_H_12_O_8_	319.04474[M-H]^-^	319.04594	193.01320,178.99731,175.00241,165.01796,125.02296	dihydromyricetin	[24, 29]
20.	30.24	C_15_H_14_O_5_	273.07678[M-H]^-^	273.07685	229.08650,189.05489,187.07535,166.02608,123.04407	epiafzelechin	[26]
21.	33.12	C_30_H_24_O_12_	575.12006[M-H]^-^	575.11950	539.09937,449.08884,423.07263,327.05060,163.00256,125.02304	proanthocyanidin A2	[25, 29]
22.	37.59	C_15_H_12_O_7_	303.05051[M-H]^-^	303.05103	285.04053,241.05025,217.04988,199.03909,177.01811,153.01816,125.02309	dihydroquercetin	[24, 25]
23.	39.39	C_35_H_60_O_6_	575.12006[M-H]^-^	575.43171	539.09747,449.08704,423.07138,327.05225,285.04007,137.02345,125.02290	daucosterol	[24]
24.	39.56	C_8_H_8_O_4_	167.03397[M-H]^-^	167.03498	123.04377	3,4-dihydroxyphenylacetic acid	-
25.	39.91	C_15_H_10_O_6_	285.04041[M-H]^-^	285.04046	241.05063,217.05078,202.02705,199.03935,175.03880	kaempferol	[24, 30]
26.	45.99	C_15_H_12_O_7_	303.05084[M-H]^-^	303.05102	151.00229,125.02303,107.01243	5,7,3',4',5'-pentahydroxydihydroflavone	[24]
27.	47.31	C_15_H_12_O_6_	287.05588[M-H]^-^	287.05611	259.06094,243.06607,201.05469,125.02309	dihydrokaempferol	[24]
28.	48.53	C_15_H_10_O_8_	317.02927[M-H]^-^	317.03029	178.99733,151.00229,137.02304,107.01238	myricetin	[24, 25, 29]
29.	54.69	C_15_H_12_O_6_	287.05609[M-H]^-^	287.05611	151.00241,135.04387,125.02351,107.01264	eriodictyol	[24]
30.	55.36	C_15_H_10_O_7_	301.03534[M-H]^-^	301.03538	178.99759,152.00613,151.00243,121.02808,107.01266	quercetin	[24, 25]
31.	58.67	C_15_H_12_O_5_	271.06076[M-H]^-^	271.06120	177.01814,151.00241,119.04889,107.01248,93.03317	naringenin	[24, 25]
32.	59.08	C_18_H_34_O_5_	329.23331[M-H]^-^	329.23335	229.14401,211.13318,183.13814,171.10132,139.11191	(15Z)-9,12,13-trihydroxy-15-octadecenoic acid	-
33.	59.21	C_15_H_10_O_6_	285.04037[M-H]^-^	285.04046	201.02451,151.00247,130.23360,106.44122	luteolin	-
34.	66.84	C_16_H_30_O_4_	285.20728[M-H]^-^	285.20713	267.19684,223.20628,158.93121	hexadecanedioic acid	-
35.	68.33	C_18_H_32_O_4_	311.22272[M-H]^-^	311.22278	293.21234,249.22192	9-HpODE	-
36.	69.50	C_18_H_34_O_3_	297.24329[M-H]^-^	297.24352	279.23334,183.13800,158.93307,91.76136	12-hydroxy-9-octadecenoic acid	-
37.	70.86	C_30_H_46_O_4_	469.33221[M-H]^-^	469.33233	436.03864,397.15903,158.92770,141.01570	29-hydroxy-3-oxotirucalla-7,24-dien-21-oic acid/Xanthocerasic acid	[29]

**Table 4:** Supplementary chemical composition of Xanthocerais lignum.

**No.**	**Compounds Name**	**References**
**Flavonoids**		
1.	rutinum	[27]
2.	cinnamtannin B1	[32]
3.	(2R,3R)-3,3',5,5',7-pentahydroxydihydroflavone	[33]
4.	epigallo-catechin-(4*β*→8,2*β*→*O*-7)-epicatechin	[34]
5.	3-methoxy-2', 4', 5, 6', 7-pentahydroxyflavone	[35]
6.	3,3′,4′,5,7-pentahydroxy-flavanone	[25, 29]
7.	gallocatechin	[25, 27]
8.	myricitrin	[25, 27]
**Triterpenoids**		
9.	3-oxotirucalla-7,24-dien-21-oic acid	[34]
10.	24-methylenecycloartan-3-ol	[34]
11.	oleanolic acid	[34]
**Phenylpropanoids**		
12.	xanthocerin	[36]
**Steroids**		
13.	stigmasterol	[37]
**Phenols**		
14.	2-hydroxy-6-methylbenzoic acid	[38]
15.	isochlorogenic acid B	[32]
16.	methyl 3-hydroxy-4-methoxybenzoate	[35]
17.	methyl 3,4-dihydroxybenzoate	[39]
18.	5,7-dihydroxychromone	[38]
**Quinones**		
19.	chrysophanol	[40]
20.	physcion	[40]
21.	emodin	[40]
22.	2,5-dimethoxy-p-benzoquinone	[40]
**Organic Acids**		
23.	(9S,10R,11E,13R)-9,10,13-Trihydroxy-11-octadecenoic acid	[24]
24.	3,4,5-trimethoxy benzoic acid	[38]
25.	nonadecanoic acid	[37]
26.	heneicosanoic acid	[37]
27.	tetracosanoic acid	[37]
**Other Chemical Components**		
28.	1,4,6,7,8-pentahydroxy-9-methyl-10-oxo-5,6,7,8,9,10-hexahydro-benzocycloocten-5-ylester	[35]
29.	dibutyl phthalate	[39]
30.	4-muurolene-3,10-diol	[39]
31.	3,4-dimethylfuran	[39]
32.	xanthocerapene	[32]

**Table 5:** Predicted results of anti-RA activity natural products in Xanthocerais lignum by integrated model.

**Target**	**Compounds (Prediction Probability: High to Low)**
PI3K	xanthocerin(25); myricitrin(15); myricetin(3); isorhamnetin-3-*O*-glucoside(16); quercetin-3-(2''-caffeylglucuronide)(18); epigallo-catechin-(4*β*→8,2*β*→*O*-7)-epicatechin(12); *β*-Sitosterol(26); cinnamtannin B1(10); rutinum(8); 3-*O*-acetyl-16alpha-hydroxytrametenolic acid(23); 24-methylenecycloartan-3-ol(21); 3-methoxy-2',4',5,6',7-pentahydroxyflavone(13); serylleucine(36); dihydromyricetin(6); stigmasterol(28); 4-muurolene-3,10-diol(34); isochlorogenic acid B(30); procyanidin A-2(9); xanthocerasic acid(19); taxifolin(5); oleanolic acid(22); (2R,3R)-3,3',5,5',7-pentahydroxydihydroflavone(11); 1,4,6,7,8-pentahydroxy-9-methyl-10-oxo-5,6,7,8,9,10-hexahydro-benzocycloocten-5-ylester(33); kaempferitrin(17); xanthocerapene(35); dihydrokaempferol(4); 3-oxotirucalla-7,24-dien-21-oic acid(20)
AKT	isorhamnetin-3-*O*-glucoside(16); cinnamtannin B1(10); procyanidin A-2(9)
Bcl2	daucosterol(27); 3,3',4',5,7-pentahydroxy-flavanone(14); 3-*O*-acetyl-16alpha-hydroxytrametenolic acid(23)
EGFR	myricetin(3); myricitrin(15); quercetin(2); rutinum(8); isorhamnetin-3-*O*-glucoside(16); quercetin-3-(2''-caffeylglucuronide)(18); kaempferol(1); epigallo-catechin-(4*β*→8,2*β*→*O*-7)-epicatechin(12); kaempferitrin(17); dihydromyricetin(6); 3-methoxy-2',4',5,6',7-pentahydroxyflavone(13); cinnamtannin B1(10)
IGF1R	myricetin(3); quercetin(2); gallic acid(31); protocatechuic acid(29); 3,4-dihydroxyphenylacetic acid(32); esculetin(24)
FAK	epicatechin-5-*O*-*β*-D-glucopyranaoside(7); rutinum(8); isorhamnetin-3-*O*-glucoside(16)
IRAK4	isorhamnetin-3-*O*-glucoside(16); daucosterol(27)

## Data Availability

The data used in this study came from public databases. The original contributions presented in the study are included in the article/supplementary material, further inquiries can be directed to the corresponding author/s.
